# A Snapshot of the Physical and Functional Wiring of the Eps15 Homology Domain Network in the Nematode

**DOI:** 10.1371/journal.pone.0056383

**Published:** 2013-02-12

**Authors:** Hanako Tsushima, Maria Grazia Malabarba, Stefano Confalonieri, Francesca Senic-Matuglia, Lisette G. G. C. Verhoef, Cristina Bartocci, Giovanni D'Ario, Andrea Cocito, Pier Paolo Di Fiore, Anna Elisabetta Salcini

**Affiliations:** 1 IFOM, Fondazione Istituto FIRC di Oncologia Molecolare, Milan, Italy; 2 Dipartimento di Medicina, Chirurgia ed Odontoiatria, Università degli Studi di Milano, Milan, Italy; 3 Istituto Europeo di Oncologia, Milan, Italy; 4 Biotech Research and Innovation Centre (BRIC), University of Copenhagen, Copenhagen, Denmark; University of Iowa, United States of America

## Abstract

Protein interaction modules coordinate the connections within and the activity of intracellular signaling networks. The Eps15 Homology (EH) module, a protein-protein interaction domain that is a key feature of the EH-network, was originally identified in a few proteins involved in endocytosis and vesicle trafficking, and has subsequently also been implicated in actin reorganization, nuclear shuttling, and DNA repair. Here we report an extensive characterization of the physical connections and of the functional wirings of the EH-network in the nematode. Our data show that one of the major physiological roles of the EH-network is in neurotransmission. In addition, we found that the proteins of the network intersect, and possibly coordinate, a number of “territories” of cellular activity including endocytosis/recycling/vesicle transport, actin dynamics, general metabolism and signal transduction, ubiquitination/degradation of proteins, DNA replication/repair, and miRNA biogenesis and processing.

## Introduction

Cellular functions are frequently carried out by large macromolecular machinery, in which proteins are assembled together through specific protein interaction modules (PIMs). In several cases, the associative potential of these modules has resulted in vast networks of interactions, such as those based on phosphotyrosine∶SH2 domains, ubiquitin∶ubiquitin-binding domains, and proline-based helices∶SH3 domains [1], [2], [3], [4], [5], [6]. Each of these networks comprises literally hundreds of proteins, thereby giving rise to thousands of protein∶protein interactions that underlie virtually every aspect of cell regulation. In other cases, PIM-based networks appear to serve more “local” purposes, in that they are specifically associated with a limited number of cellular functions. In this latter instance, one might hypothesize that these networks evolved to connect different functional “territories” of cellular activities, whose operations need to be coordinated for the execution of certain cellular processes. The deconvolution of the complete physical and functional wiring of these “local” networks is facilitated by their limited extension, and can potentially reveal elements of the higher level of organization and hierarchy of basic cellular functions.

The EH-network represents a case in point [7]–[16]. This network is established through the EH (Eps15 Homology) domain, a protein∶protein interaction module originally identified, in three copies, in the endocytic proteins eps15 and eps15R [11]. A variety of approaches identified three classes of EH-binding peptides [9], [10], [17]–[19]. The majority of EH domains bind preferentially to NPF (asparagine-proline-phenylalanine)-containing peptides (class I peptides), or to variants thereof (DPF- or GPF-containing peptides) [9], [10], [17]–[25]. In keeping with these results, several proteins that specifically interact with EH domains have been identified; all possess NPF motifs (see for instance [10], [26]–[41]. Two other classes of EH-binding peptides are known, class II (FW, WW or SWG di- or tri-peptides) and class III (HSF and HTF tripeptides), although it is not completely clear whether these motifs represent true physiological binders or peptidomimetics [9], [10], [17]–[19]. EH domains are also able to bind to phosphatidylinositols [42]–[44].

One appealing feature of the EH-network is its limited size. There are eleven EH-containing proteins in the human genome, grouped into 5 families, and these are conserved from nematodes to mammals [8]. The domain is also present in yeast (discussed below). Many studies have been directed at understanding the physiological role(s) of the EH network [7], [8]. The combined analysis of the properties of EH-containing proteins and of the cellular proteins that interact with them allows us to extrapolate some general concepts, which point to the EH-network as an integrator of signaling pathways. First, the majority of the EH-network proteins have established functions at various steps of the endocytic route and in the process of synaptic vesicle recycling [7], [8], [45]. Second, some EH-network proteins participate in other events of intracellular traffic, for example, γ-synergin is involved in Golgi to endosome trafficking [46]. Third, EH-network proteins are also involved in the organization of the actin cytoskeleton [7], [8], [45]. Finally, a number of EH-containing and EH-interacting proteins shuttle in and out of the nucleus [32], [47]–[49], where they might participate in the control of transcription or of other nuclear events [7], [8], [45], [50]. In summary, the EH network appears to integrate several physiological functions and its subversion is involved in relevant pathological conditions, including cancer [51]–[53].

The limited extension of the EH-network makes it an attractive protein∶protein network for high-resolution physical and functional mapping at an organismal level. We chose the nematode *C. elegans* as a model system because, in addition to its genetic tractability, which is paramount for functional studies, *C. elegans* possesses only five EH-containing proteins, representative of each of the five mammalian EH families: the Eps15, Intersectin, EHD, Reps and γ-synergin families (Figure S1). Thus, the nematode EH-network can be considered a simplified “prototypical” version of its mammalian counterpart. Lower organisms, such as *S. cerevisiae*, do not possess all orthologues of mammalian EH-containing proteins (Figure S1), thus reinforcing the idea that *C. elegans* is the simplest model system that can be used to obtain information that can be extrapolated to mammalian physiology. In this paper, we report the physical and functional wiring of the EH network, at the organismal level, in the nematode.

## Materials and Methods

### Material

All chemicals were obtained from Sigma-Aldrich unless otherwise specified. Actin mAb was from Biomedicals, FLAG pAb from Sigma-Aldrich and GFP mAb from Roche.

### Yeast Two Hybrid

The Yeast Two Hybrid screen was performed according to the ProQuest™ Two-Hybrid system Instruction Manual (Invitrogen). Regions containing the EH domains of *ehs-1* (aa 1–329 and aa 254–430) and *itsn-1* (aa 1–264) were obtained by recombinant PCR using specific ESTs kindly provided by Dr. Yuji Kohara from the *C. elegans* consortium. Regions containing the EH domains of *rme-*1 (aa 668–786), *reps-1* (aa 1–213) and *R10E11.6* (aa 55–360) were amplified from a *C. elegans* cDNA library. Sequences of the primers used in the amplification procedure are available upon request. EH-containing regions were cloned in the pDBLeu vector and tested for self-activation using *LacZ* expression before use. Appropriate 3-Amino-1,2,4-Triazole (3-AT) amount was added to titrate the minimum level of Histidine expression required for selection by growth on Histidine-deficient media of the co-transformants.

The *C. elegans* cDNA library (pPC86-cDNA library) was purchased from Invitrogen. 10 µg of bait and 10 µg of *C. elegans* cDNA library were co-transformed in MaV203 competent cells and plated in selective medium. For each bait 10^6^ colonies were screened to ensure that the complexity of the whole genome represented by the cDNA library was covered. Positive clones were selected for growth in selective media and for *LacZ* expression. Yeast DNA was extracted, transformed in *E. coli* and sequenced using a specific oligo for the prey vector pPC86 (5′ TATAACGCGTTTGGAATCACT 3′). The cDNA inserts identified were re-transformed with the specific bait into MaV203 competent cells and the re-transformants were tested for growth in selective media and for the expression of *LacZ*. Theoretical binding partners such as UNC-11, SCM-1 FBXB-75, R06F6.2 and UNC-26 were cloned in the prey vector pPC86 and tested for interaction in a similar set-up.

### Quantitative PCR

**Figure 1:**
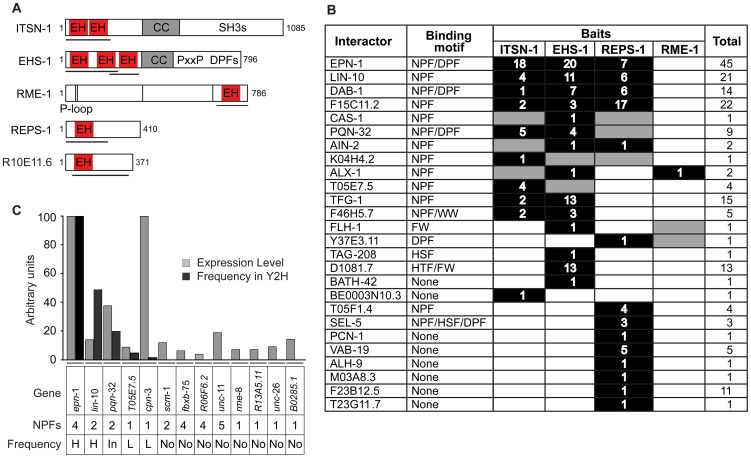
Yeast Two Hybrid analysis of EH-proteins in *C. elegans*. (A) Schematic diagram of the five EH-containing proteins in *C. elegans*. Note that several isoforms are reported in wormbase. Here, we show the isoforms cloned, sequenced and used for the described experiments. Baits used for the Y2H are indicated by black lines. For EHS-1, two distinct baits were used in the screens, since a bait spanning the three EH domains showed self-activation. CC, coiled-coil region; SH3, region containing multiple SH3s in ITSN-1; PxxP, region containing multiple SH3-binding sites in EHS-1; DPFs, region containing multiple AP-2-binding sites in EHS-1; P-loop, nucleotide-binding domain in RME-1. (B) Results of the Y2H screen. The 26 identified EH-interactors are listed. Potential EH-binding motifs are indicated. Black, interactions detected in the initial screen; gray, interactions detected in the re-transformation assay (see text). The number of clones identified in the initial screen is also shown. No interactions were detected for R10E11.6. (C) The indicated genes were tested by quantitative PCR in the yeast library used for the Y2H screening. The number of EH-interacting motifs (NPF) and the frequency of identification in the Y2H (H, high; In, intermediate; L, low; No, no interaction) are shown at the bottom. The estimated number of copies present in the cDNA library is shown, by grey bars, in arbitrary units relative to the level of representation of *epn*-1 that was set to 100. As a comparison we show, using black bars, the frequency of isolation of the various clones in Y2H, again relative to the frequency of isolation of *epn*-1 that was set to 100 ( = 45 clones).

The cDNAs of selected genes indicated in Figure 1C were amplified from the cDNA library with specific oligos (sequences are available upon request) and the number of copies present in the cDNA library was quantified by quantitative PCR (qPCR) using SYBR Green (Applied Biosystems) in an ABI Prism 7700 Real Time PCR system.

### Validation by *in vitro* binding assays

Full-length cDNAs encoding for *C. elegans* EHS-1 (aa 1–796), ITSN-1 (aa 1–1085), RME-1 (aa 1–786) and REPS-1 (aa 1–410) were cloned in pCDNA vector in frame with a FLAG tag. The LWA mutants, used for the experiments depicted in Figure S3, were obtained by site-directed mutagenesis. Two residues, a Leucine and a Tryptophan whose positions are indicated in Figure S2, that are highly conserved and critical for the binding abilities of EH domains, were mutagenized to Alanine. All the EH domains, contained in the various proteins, were mutagenized. Thus, the EHS-1^LWA^ mutant (3 EH domains) harbors 6 mutations to Alanine; the ITSN-1^LWA^ mutant (2 EH domains) harbors 4 mutations to Alanine; the REPS-1^LWA^ and RME-1^LWA^ mutants (1 EH domain each) harbor 2 mutations to Alanine each. EHS-1, ITSN-1, RME-1 and REPS-1, WT or LWA mutant, were expressed in Phoenix cells by transient transfection. Expression of the proteins was verified by immunoblot using anti-FLAG antibody.

Y2H positive cDNA inserts were subcloned in pGex-6P-2 vector (if different lengths of DNA inserts for the various interactors were available, the shortest insert was chosen) and transformed in *E. Coli BL21* strain. Bacteria were induced with 1 mM IPTG for 5 hours at 30°C and the purification of GST proteins was performed using Glutathione Sepharose 4B beads according to manufacturer's instructions (Amersham-Pharmacia Biotech).

*In vitro* binding assays were performed incubating for 2 hours 10 µg of GST proteins with lysates harboring the *C. elegans* EH-containing proteins prepared in JS buffer (Hepes 50 mM pH 7.4, NaCl 150 mM, Glycerol 10%, Triton ×100 1%, MgCl_2_ 1,5 mM, EGTA 5 mM, Protease Inhibitor cocktail set III EDTA free from Calbiochem). Beads were washed three times with JS buffer and the proteins eluted in SDS buffer were loaded in SDS-PAGE gels. Immunoblots were performed using anti-FLAG antibody and the results of at least three independent experiments were analyzed using ImageJ program.

### *C. elegans* methods

*C. elegans* strains were cultivated using standard conditions [54] The Bristol strain (N2) was used as the WT strain. Other strains used were: *ehs-1(ok146)*, *itsn-1(ok268)*, *rme-1(b1045)*, *reps-1(tm2156)*. The *reps-1(tm2156)* strain was generated by Shohei Mitani of the National BioResource Project, Tokyo Women's Medical College (Tokyo, Japan) and was outcrossed four times with N2 before phenotypic analysis. The *reps-1* locus in the *tm2156* allele was sequenced to confirm the deletion annotated in Wormbase.

RNA interference was performed as described [55], using clones obtained from the RNAi feeding library construct generated by the J. Ahringer's laboratory (J. Ahringer, Wellcome Trust/Cancer Research UK Gurdon Institute, University of Cambridge, Cambridge, UK). Clones used for the RNA interference were sequenced before use. Synchronized L1 larvae, obtained by hypochlorite treatment of gravid adults, were added to the feeding plates and incubated at 15°C until they reached young adulthood.

For the aldicarb assays, plates were prepared adding aldicarb (Chem Service, West Chester, PA) solution (in 70% ethanol) to the agar prior pouring the plates. Aldicarb plates were seeded with OP50 bacteria and freshly used. Twenty or thirty young adult worms of each strain were transferred from RNAi feeding plates onto aldicarb plates, in duplicate, and each worm was tested for touch response using the tip of the platinum rod every 30 minutes for 3 hours, and the number of worms that responded to touch was recorded. The assay was repeated at least twice, testing RNA interfered worms generated by independent RNAi experiments. A similar experimental setting was used to score the response to aldicarb of *ehs-1*, *itsn-1* and *reps-1* mutant strains.

The *REPS-1p::REPS-1::GFP* construct was generated by two cloning steps. Firstly, 3609 bps of the *reps-1* promoter region were PCR-amplified from N2 genomic DNA and cloned into the SalI-XmaI restriction sites of the pPD95.75 vector (Fire lab) to generate the construct *REPS-1p::GFP*. Primers used were:

5′-AT**CCCGGG**GTTCTGTCATGGAAATTGATTTTTTCGCG-3′

5′-CACA**GTCGAC**GTCATTCGAATATCGCTTC-3′

Secondly, a 4548 bp fragment, containing the *reps-1* locus, was PCR-amplified from N2 genomic DNA and cloned into the BamHI-SmaI restriction sites of the *REPS-1p::GFP* construct thus generating the *REPS-1p::REPS-1::GFP* construct. Primers used were:

5′-GTCGGT**GGATCC**GAATCGAATCCGCTGC-3′

5′- AT**CCCGGG**GAAGTGTAGAAGAAGAGCACGC-3′

To obtain lines carrying extra-chromosomal arrays, the *REPS-1p::REPS-1::GFP* construct (15 ng/µl) was co-injected with ttx-3::DsRed construct as injection marker (100 ng/µl) in wild-type N2 worms. Several transgenic lines were generated and analyzed for level of expression and localization of the transgene. Pictures of transgenic animals anesthetized with 2 mM levamisole were acquired using an Axiovert 135, Carl Zeiss, Inc.

### Statistical analysis

The data collected from the aldicarb assay were subjected to statistical analysis in order to score genetic interactions. Statistical significance was analyzed by the method described below, implemented on a dedicated software developed in-house. The time needed to develop an aldicarb response (“time to immobilization”) by each group of animals was modeled as a two-parameter Weibull cumulative distribution function: 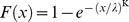. The value of the *k* parameter (shape) was estimated globally at the least squares, resulting to be *k = 2.5*. For each experiment and condition, the scale parameter (*lambda*) was estimated by means of a Levenberg-Marquardt algorithm; the confidence interval for the estimated *lambda* was computed in the four conditions (WT, KO strain, RNAi in WT, RNAi in KO strain) as described [56] with a simplified Gauss-Newton method. The log ratio of the “time to immobilization” for the perturbed conditions with respect to the WT was then computed using confidence range propagation; changes with respect to WT were finally averaged among the replicated experiments, obtaining the global confidence interval of *lambda* for each condition. The null hypothesis was assumed to be a simple cumulative effect on “time to immobilization” expectation of the “KO strain” and “RNAi in WT conditions”. A genetic interaction was scored when the observed “time to immobilization” of the “RNAi in KO strain” condition differed significantly (p<0.05) from the null hypothesis.

## Results

### Identification of EH interacting proteins by Yeast Two Hybrid screening

Four of the five families of EH-containing proteins are represented in *C. elegans* by a single gene: eps15/*ehs-1*, intersectin/*itsn-1*, EHDs/*rme-1*, REPS/*reps-1*, as shown in Figure 1A. In addition, an uncharacterized gene, R10E11.6, shows homology to γ-synergin, and was therefore included in our screening (Figure S1). The EH domains of the five EH-containing proteins were cloned and used as baits to screen a *C. elegans* cDNA library, prepared from a mixed population of all developmental stages, by the yeast two hybrid methodology (Y2H).

Twenty-six proteins interacted with at least one of the baits; frequently the same proteins interacted with more than one bait, and were isolated multiple times (Figure 1B). To better understand the specificity of the interaction of each EH-containing protein, all the 26 interactors were re-tested, by Y2H, against all of the baits, allowing the identification of a few additional interactions (shown in grey in Figure 1B). From the complete matrix of interactions a number of features of the EH-network emerged: i) around half of the EH-binding proteins interacted with more than one EH-containing protein (14 of 26, “promiscuous interactors”); ii) The remaining 12 of 26 EH-binding proteins, conversely, displayed binding selectivity for one of the baits (“specific interactors”); iii) the “promiscuous interactors” displayed, in the large majority of cases (12 of 14), canonical class I NPF motifs; iv) on the other hand, “specific interactors” contained NPF motifs only in 2 cases, and in the majority of cases (9 of 11) they did not harbor any known EH-interacting sequence; v) NPF-containing proteins represented ∼54% of the interactor pool (14 of 26 proteins), but accounted for ∼80% of all identified clones (148 of 186), suggesting that NPF-mediated interactions are probably stronger and more stable than other interactions (see additional controls below); vi) the EH domains of ITSN-1 and EHS-1 displayed remarkably overlapping binding abilities (12 common interactors out of 13 and 16 interactors, respectively); vii) the EH domain of REPS-1 displayed two types of binding, to promiscuous interactors (almost invariably in common with both ITSN-1 and EHS-1) and to specific interactors; viii) the EH domain of RME-1 displayed the highest level of selectivity, binding to only 3 proteins (all promiscuous interactors); ix) the EH domain of R10E11.6 did not show any interaction.

This latter finding deserves additional comments. R10E11.6, is a candidate homologue of mammalian γ-synergin. It is of note that binding partners for rat γ- synergin could not be identified by several methods (Y2H, GST pulldown, overlay experiments [46]). However, SCAMP1 – a membrane-associated protein – was shown to bind to rat γ-synergin, in an NPF-motif dependent manner [31], suggesting a canonical EH∶NPF interaction together with a rather narrow specificity. We directly tested, by Y2H, whether the nematode homologue of SCAMP-1, SCM-1, could bind to the EH domain of R10E11.6, but detected no interaction (not shown). This result, together with the lack of any interaction in the Y2H screening argues that the putative EH domain of R10E11.6 is not a true EH domain. In support of this possibility, we note that the EF-hand motif, found in almost every EH domain [17], is missing in R10E11.6; furthermore a proline residue is present in the loop connecting helices three (H3) and four (H4), both of which are critical for the EH structure. Thus, a rigid bond in the loop connecting H3 and H4 might deform the structure of the EH domain in this critical region, possibly reducing the affinity of this particular EH domain to NPF containing peptides (see Figure S2).

Finally, we performed a number of control experiments to verify that the list of EH-interactors derived from the Y2H screening constituted a reliable representation of the EH interactome in the nematode. First, we wanted to exclude that the frequency of isolation of the clones was simply a reflection of their abundance in the cDNA library. We also wanted to verify whether other potential interactors (for instance proteins harboring multiple NPF motifs) were not isolated simply because of their lack of representation in the library. Thus, we performed quantitative PCR to test the level of representation of a number of genes in our cDNA library. A total of 13 genes, listed in Figure 1C, were selected for this analysis. We chose genes encoding NPF-containing proteins identified in the Y2H screenings at high (*epn-1*, *lin-10*), intermediate (*pqn-32*), or low frequency (*T05E7.5*). We also included *C. elegans* genes encoding proteins not identified in the screen which might in principle display EH-binding activity based on results obtained in other organisms (hypothetical partners, *unc-11*, *unc-26*, and *scm-1*), or because they contained multiple (*fbxb-75* and *R06F6.2*) or single NPF motifs (*rme-8*, *cpn-3*, *R13A5.11*, and *B0285.1*). There was no correlation between the levels of expression of the 13 genes and the frequency of their detection in the Y2H screening (Figure 1C). In particular, a number of cDNAs, whose encoded products were not detected in the screening [such as the hypothetical partners *unc-11*/AP180 [57], [58] and *scm-1*/SCAMP-1 [31], or proteins with several NPF repeats, e.g. FBXB-75 and R06F6.2/*vps-11*], were expressed at levels similar to those of cDNAs whose proteins were reproducibly detected in the screening (such as *lin-10* or *T05E7.5*) (Figure 1C). As a final control, UNC-26, a nematode homologue of Synaptojanin not detected in our screening – which in mammals interacts with Eps15 and Intersectin [27], [29] –, was cloned in-frame with GAL4AD and tested for interaction with ITSN-1, EHS-1, RME-1 and REPS-1 in Y2H. None of the re-transformed clones showed *LacZ* expression (not shown), indicating a lack of interaction, at least under our experimental conditions.

We concluded, that the Y2H screening yielded a reliable representation of the EH interactome in the nematode.

### Validation of EH-interacting proteins by *in vitro* binding assay

The interactions identified by Y2H were further validated by *in vitro* pull-down assays. Sixteen of 26 EH-binding proteins were selected to represent a range of EH-interacting motifs found in the protein sequences. The shortest cDNA identified in the Y2H screening for each EH-interactor was expressed as a GST-fusion protein. The *C. elegans* EH-containing proteins were over-expressed as FLAG-tagged full-length proteins in Phoenix cells (not shown), and total cellular lysates were used as a source of EH-containing proteins for *in vitro* pull-down experiments. Full-length EH-containing proteins were used, in order to obtain proteins as close as possible to their native state, and also to facilitate interactions that might be mediated by the EH domains but assisted by other regions of the EH-containing proteins, as has been shown to be the case for the binding partners of some EHD family proteins [39], [59] .

**Figure 2:**
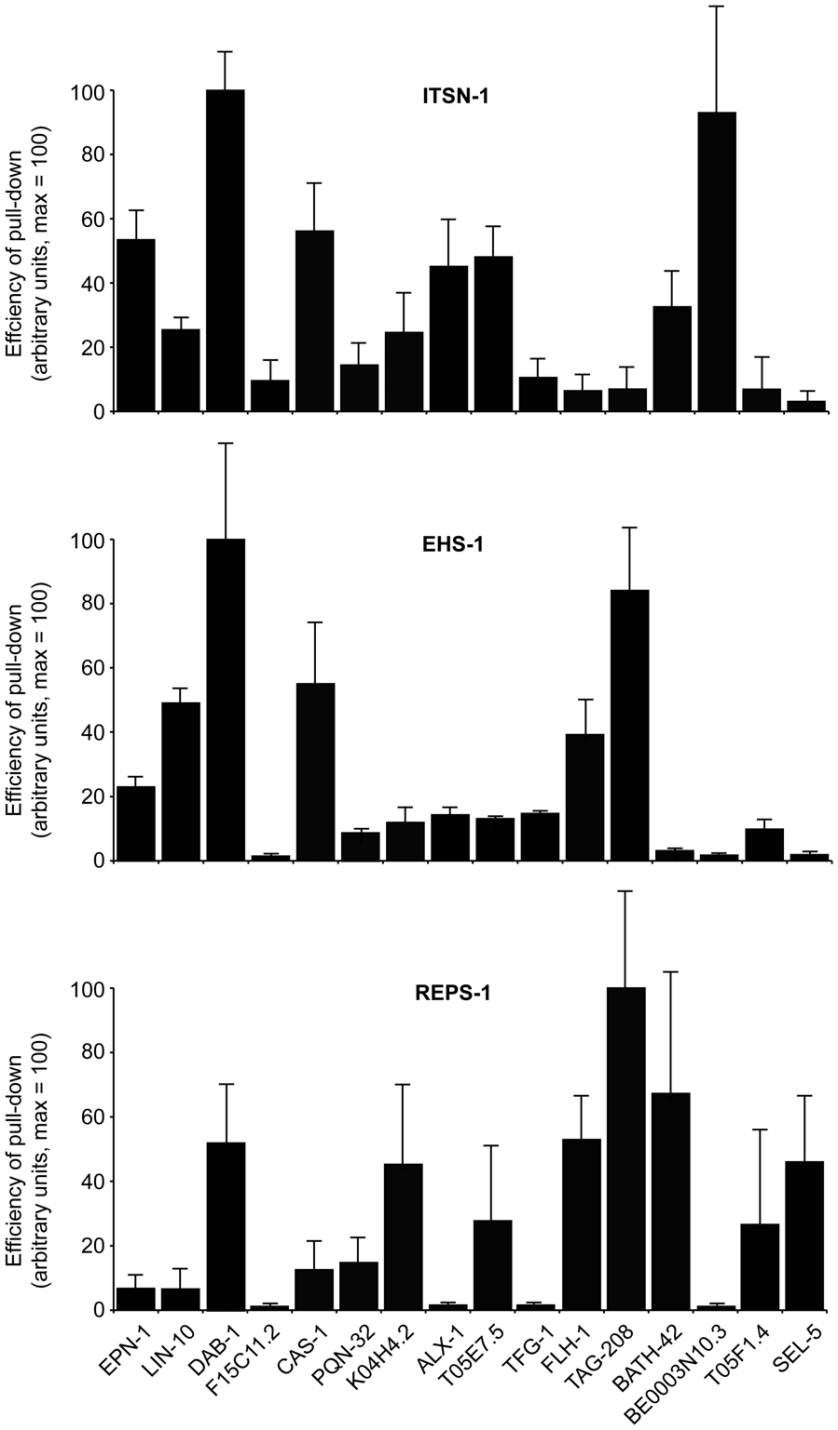
In vitro binding assays. Sixteen interactors, identified by Y2H (listed at the bottom), were expressed as GST-fusion proteins and used for *in vitro* binding assays with FLAG-EH proteins expressed in Phoenix cells. Results are the average of three independent experiments (examples are shown in Figure S3), and are expressed in arbitrary units on a scale 0–100, in which 100 represents the efficiency of the pull-down for the strongest interacting protein in each panel.

In Figure 2, we report the average results of several independent determinations (at least three for each EH-protein∶EH-interactor pair), for ITSN-1, EHS-1 and REPS-1 (see also Figure S3, for examples of the actual blots). In general, the results of the *in vitro* binding assays agreed well with those of the Y2H screening. Of 48 possible combinations, 37 (∼78%) were concordant between the two assays (see Figure S4 for a synopsis of the results). Importantly, some of the “selective interactions” were validated in the *in vitro* binding assay. For example, this is the case for BE0003N10.3, which specifically interacted with ITSN-1, and of SEL-5, which displayed preference for REPS-1 (see Figure S4). The preference of FLH-1 and of TAG-208 for EHS-1, but not for ITSN-1, was also confirmed. These latter two proteins also interacted with REPS-1 in the *in vitro* binding assay, but not in the Y2H assay (although the interaction of REPS-1 with TAG-208 does not appear to be directly mediated by the EH domain of REPS-1, see below). While we have no immediate explanation for this (and other discrepancies), it is important to note that indirect interactions (for instance through dimerization with other EH-containing proteins, a case well established – for instance – for EHS-1/eps15 and ITSN-1/Intersectin) are more likely to occur in an *in vitro* binding assay than in a Y2H assay, given the design of our experiments. Finally, some interactors identified by the Y2H assay were not confirmed by the *in vitro* binding assay, as is the case for the binding of EHS-1 and REPS-1 to F15C11.2, or the binding of EHS-1 to BATH-42. One obvious possibility is that, in some cases, interactions evidenced by the Y2H represent false positives. This is a well-recognized problem with this kind of assay, possibly due to the fact that interacting proteins are abundantly co-expressed in the nucleus of the yeast. While this caveat must be acknowledged, it appears to affect a minority of the interactions herein reported. Another possibility is that some of the GST fusion proteins, used in the *in vitro* binding assays, might not be properly folded. F15C11.2 might represent a case in point, as this protein – in the GST configuration – was overall a weak binder. It is worth noting that F15C11.2 is the homologue of human ubiquilin, a protein that has been reported to interact with the mammalian EHS-1 homologue, Eps-15 [60], suggesting that some the interactions identified by H2Y, but not further validated possibly for technical reasons, may instead be genuine.

A separate analysis is needed for the results obtained with RME-1. In the Y2H assay, we detected only three interactors for the EH of this protein: one in the original screening (ALX-1) and two (FHL-1 and Y37E3.11) in the subsequent re-validation with the entire pool of EH interactors. This in principle might mean that our bait was not adequate (e.g. improperly folded) or that the EH domain of RME-1 is a weak interaction surface that needs contributions from other regions of the protein to establish detectable interactions. For this reason, we performed *in vitro* binding assays not only with full length RME-1, but also with a mutant in which two point mutations, in highly conserved residues [9], were introduced to abolish the binding properties of the EH domain (see Figure S2 for the position of the mutagenized amino acids). The results in Figure S3 show that many of the EH interactors could bind to RME-1 efficiently. However, the presence of the EH domain contributed to the interaction only in a few cases (as witnessed by decreased binding to the EH mutant RME-1^LWA^). While we do not know whether the detected interactions are direct or indirect, these results suggest that the EH domain of RME-1 *per se* is a weak protein∶protein interaction surface that may require other elements to acquire binding specificity, as further discussed below. This latter result prompted us to further ensure that the identified interactions for EHS-1, ITSN-1 and REPS-1 were EH-dependent. To this end, we performed *in vitro* binding experiments using mutated versions of these proteins, in which all the EH domains were mutagenized with point mutations similar to the RME^LWA^ (see Figure S2 for the position of the mutagenized amino acids). As shown in Figure S3, the majority of the interactions was lost when the EH domains of these proteins were mutagenized, indicating their relevance in the identified interaction. A notable exception was represented by the interaction between TAG-208 and REPS-1, which was not appreciably affected by the presence of mutations in the EH domain of REPS-1, thus indirectly confirming the absence of interaction between these two proteins in the Y2H assay.

### *reps-1* is ubiquitously expressed and has a role in neurotransmission

As an important part of our attempt to obtain the complete physical and functional wiring of the EH network in nematode, we wanted to perform functional studies of the interactions between EH-containing and EH-binding proteins, by exploiting the power of reverse genetics in *C. elegans*. Three of the four EH-containing nematode proteins and genes, EHS-1, ITSN-1, and RME-1 have been previously characterized at high resolution [61]–[64]. However, REPS-1 and its gene, *reps-1*, remain uncharacterized. Thus, we therefore performed a preliminary characterization of REPS-1.

**Figure 3:**
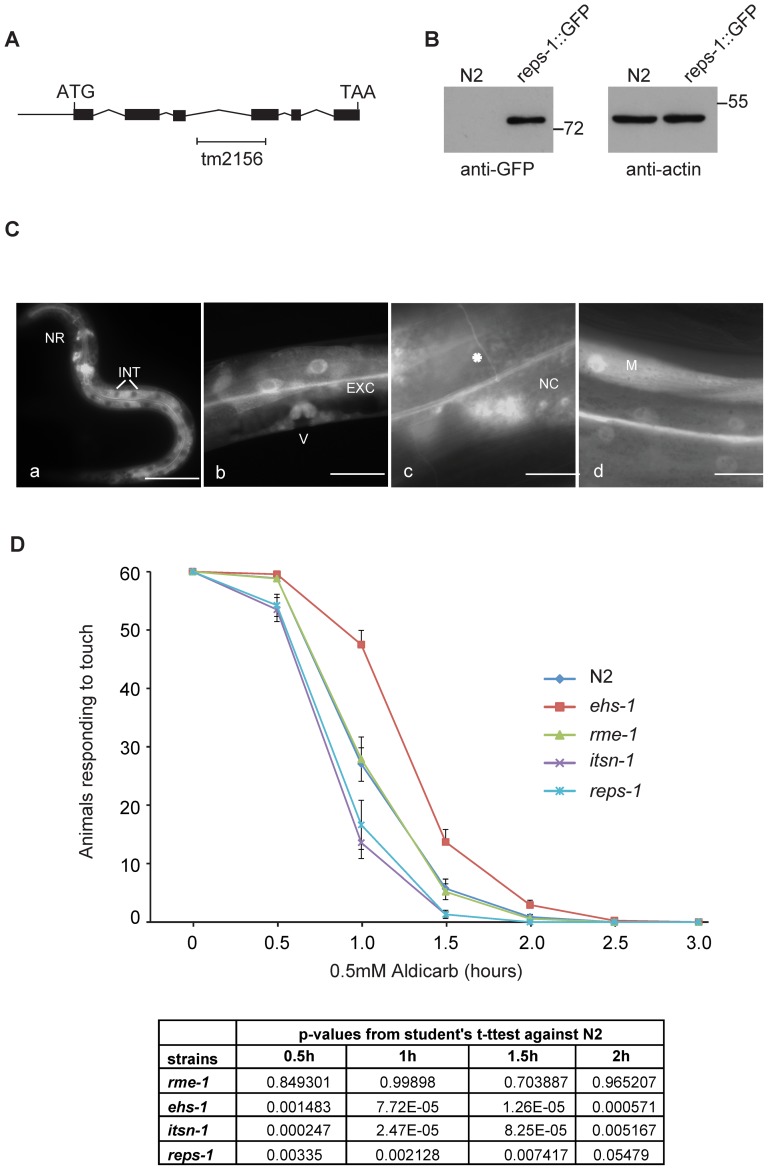
REPS-1 expression and function. (A) The *reps-1* locus. The deletion in the *tm2156* strain is also shown. (B) Protein lysates from wild-type and a transgenic line carrying a translational fusion of the *reps-1* gene with GFP (*reps-1p::REPS-1::GFP*) were probed with indicated antibodies. (C) Images (epifluorescence) of hermaphrodites carrying the *reps-1p::REPS-1::GFP* transgene. Anterior is to the left, ventral down. NR: nerve ring, INT: intestine, ESC: excretory system, V: vulva, NC: nerve cord, M: muscle cell. The asterisks indicate nerve commissures. Bars: 100 µm in a, 20 µm in b, 10 µm in c and d. (D) Aldicarb test on strains carrying mutations in EH proteins. Synchronized young adult animals were plated onto NGM plates containing 0.5 mM aldicarb and assayed after the indicated times for movement. The number of animals responding to a light touch with a platinum wire is reported. Results are the average of three independent experiments, each performed on 60 animals/strain. Note that the curves for N2 and *rme-*1 worms overlap almost completely. P values are indicated in the table.

A mutant strain, *reps-1(tm2156)*, was obtained from the National Bioresearch Project (Japan). *reps-1* is predicted to encode for a protein of 410 amino acids and its genomic organization is presented in Figure 3A. The *tm2156* mutant allele has a deletion of 779 bases resulting in loss of the third intron and of a portion of the fourth exon. *reps-1(tm2156)* animals appear to be wild type at different temperatures, in terms of viability, fertility and locomotion (not shown). To gain insight into *reps-1* functions, we analyzed its expression pattern using transgenic lines carrying the *reps-1* gene under its own promoter, in fusion with a GFP reporter. The expression of the fusion protein was analyzed in lysates of transgenic worms by western blot analysis, revealing a protein band with an apparent molecular weight of 75 kDa, in agreement with the predicted molecular weight for REPS-1::GFP (Figure 3B). The transgenic lines showed expression in many tissues including intestine, secretory system, vulval cells and muscle cells (Figure 3C). REPS-1 was also expressed in the nervous system with diffuse staining in the nerve ring, ventral cord and commissures, but no expression was observed in the neuronal body (Figure 3C). When tested for sensitivity to aldicarb, an inhibitor of acetylcholine esterase often used to reveal defective cholinergic transmission, the *reps-1* mutant showed an abnormal response, with hypersensitivity to the drug compared to wild type animals, a phenotype reminiscent of that detected in *itsn-1*-null nematodes [62] (Figure 3D). The aberrant response to aldicarb that may be related to deficiencies at neuronal and/or muscular levels, where REPS-1 is expressed (Figure 3C), strongly suggests a role of REPS-1 in neurotransmission. This result does not exclude, obviously, other possible functions for REPS-1, as also suggested by the wide pattern of expression of the gene.

Whatever the case, however, the aldicarb phenotype provides a bioassay for the further characterization of genetic interactions in *reps-1(tm2156)* animals.

### Genetic interactions within the EH network

*C. elegans* mutant alleles for the four *bona fide* EH-containing proteins (EHS-1, ITSN-1, REPS-1, and RME-1) are available as viable strains. In the nematode, eps15/EHS-1 and intersectin/ITSN-1 are implicated in synaptic transmission and regulate dynamin function and localization during synaptic vesicle recycling [61]–[64]. EHS-1, ITSN-1 and REPS-1 are all expressed in the nervous system and their functions are revealed by aberrant aldicarb sensitivity with *ehs-1*-null animals displaying resistance, and *itsn-1*-null and *reps-1*-mutant animals displaying hypersensitivity to aldicarb, respectively (Figure 3D and [61]–[64]). *rme-1* null mutant animals show, conversely, a wild-type response to aldicarb (Figure 3D). Therefore, to uncover genetic interactions within the EH network, we concentrated on aldicarb-sensitivity assays, which in principle could reveal such interactions between EH-binding proteins and three of the four EH-containing proteins (EHS-1, ITSN-1, and REPS-1).

**Figure 4:**
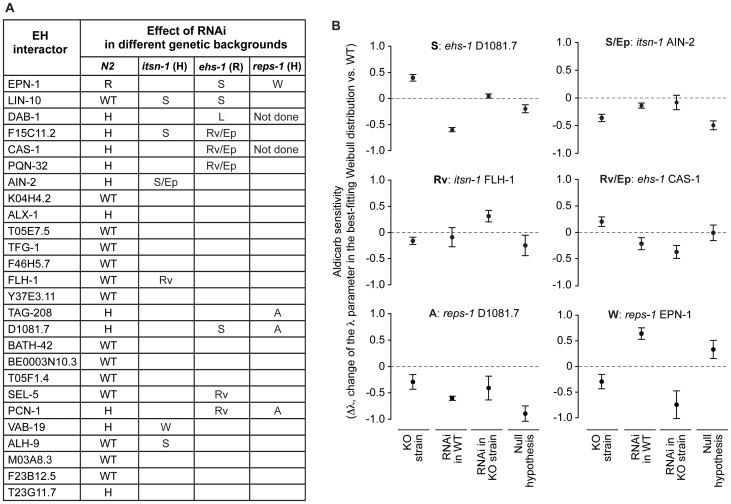
Effect of RNAi of EH interactors in various genetic backgrounds. Down-regulation of the EH-interactors was achieved by feeding RNA interference (RNAi), in the indicated strains, and animals were tested for aldicarb sensitivity. (A) In the column N2, the effect of RNAi on aldicarb sensitivity in wild type (N2) animals is reported (H, hypersensitive to aldicarb, R, resistant to aldicarb). In the other columns, the type of genetic interaction, detected in the various strains, is reported (S, suppressing; W, worsening; Rv, reverting; A, asynthetic; L, lethal; Ep, possibly RNAi epistatic; see also Table 1). (B) Examples of the detected genetic interactions. Results are expressed as the change in the λ parameter in the best-fitting Weibull distribution with respect to WT. “KO strain”, null mutant for the EH-containing gene; “RNAi in WT”, N2 worms in which the EH-interactor was silenced by RNAi; “RNAi in KO strain”, null mutants for the EH-containing gene in which the EH-interactor was silenced by RNAi; Null hypothesis, mathematical sum of the observed phenotypes in the “KO strain” and “RNAi in WT conditions”. Details of the analysis are in Materials and Methods.

Initially, we analyzed the effect of RNAi of the various EH-interactors on aldicarb sensitivity. The expression of the EH-interactors was knocked down (KD) in wild type N2 (WT) animals through feeding RNA interference (RNAi), and the resulting phenotypes were analyzed as described in Materials and Methods. In several instances (11 of 26 genes), we detected an aldicarb hypersensitive phenotype in N2 animals (Figure 4A); in the case of the *epn-1* gene, the interfered worms displayed an aldicarb-resistant phenotype (Figure 4A). These results are consistent with an important involvement of the EH network in neurotransmission, albeit with the caveat that an aldicarb-hypersensitive phenotype might also derive from more general effects of individual KDs, which could result in unhealthy animals that might be more sensitive to the drug, independently of neurotransmission defects. However, RNA interfered animals did not show any apparent phenotypes or signs of sickness, apart for DAB-1 KD animals, that showed molting and egg laying defects, as already reported [65], thus favoring the notion that our results are indeed directly linked to neurotransmission defects.

We then tested for genetic interactions, by performing RNAi of the various EH-interactors in the *ehs-1*, *itsn-1* and *reps-1* mutant genetic backgrounds. We recorded aldicarb phenotypes in four different conditions: i) “WT”, set as baseline for normalization, ii) “KO strain” (either *ehs-1*, or *itsn-1*, or *reps-1* mutants), iii) RNAi of individual EH-interactors in the WT (N2) background (“RNAi in WT”); iv) RNAi of individual EH-interactors in the various EH-mutant backgrounds (“RNAi in KO strain”). Genetic interactions were scored when the aldicarb-response phenotype of “RNAi in KO strain” was statistically different (p<0.05) from the sum of the individual phenotypes of the “KO strain” and of the “RNAi in WT” conditions (thus assuming a mere additive effect as the null hypothesis). The various types of genetic interactions (suppressing, reverting, worsening) were named according to the effect that silencing of the EH-interactor gene had on the aldicarb response of the EH-mutant strain, by comparing the “RNAi in KO strain” to the “KO strain” conditions (see also Table 1). Finally, we also annotated when the RNAi of the EH-interactor seemed to produce a dominant phenotype in a given KO strain (possibly RNAi epistatic) and when the conditions “KO strain”, “RNAi in WT” and “RNAi in KO strain” showed indistinguishable phenotypes (asynthetic). All the results are shown in Figure 4A, and examples of the actual data are given in Figure 4B. In summary, a number of EH-interactors (14 of 26) displayed genetic interactions with at least one EH-encoding gene, thus indicating functional links.

**Table 1:** Description of genetic interactions.

Type of interaction	Description and observed phenotype
Suppressing	RNAi of the EH-interactor in the KO strain causes an amelioration of the aldicarb response with respect to the KO strain.
Reverting	RNAi of the EH-interactor in the KO strain causes an opposite aldicarb response with respect to the KO strain.
Worsening	RNAi of the EH-interactor in the KO strain causes a worsening of the aldicarb response with respect to the KO strain.
Lethal	RNAi of the EH-interactor (RNAi) in the KO strain causes lethality. Double mutant animals died at L2–L3 larval stages.
Possibly RNAi epistatic	RNAi of the EH-interactor (RNAi) in the KO strain seems to mask the aldicarb response with respect to the KO strain. Double mutant animals showed an aldicarb phenotype similar to that observed in RNAi treated animals.
Asynthetic*	Gene silencing of the EH-interactor (RNAi) in wild type, EH mutant strains (KO strain), as well as double mutant animals show comparable aldicarb response.

Aldicarb sensitivity was measured, as described in Materials and Methods, at 0.5 mM aldicarb. A genetic interaction was scored when the aldicarb-response phenotype of the condition “RNAi in KO strain” was statistically different (p<0.05) from the sum of the individual phenotypes in the conditions “KO strain” and “RNAi in N2” (null hypothesis). The type of genetic interaction was further defined according to the effect that silencing of the EH-interactor gene had on the aldicarb response of the EH-mutant strain, by comparing the “RNAi in KO strain” to the “KO strain” conditions, as specified in the Table.

* as defined by Drees et al. [97].

## Discussion

**Figure 5:**
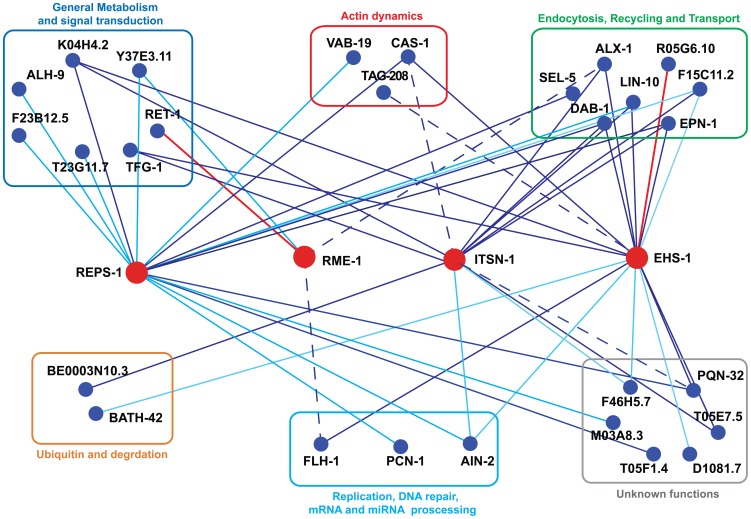
The EH network in *C. elegans*. An interaction diagram is shown representing *C. elegans* EH proteins (red circles) together with their interactors (blue circles); the interactors are further grouped into functional categories that were derived from the Wormbase and the Gene Ontology databases, from the literature, or inferred from functions of the mammalian homologues. Interactions uncovered in this study by Y2H are shown by light blue lines. Interactions confirmed by *in vitro* binding assays are shown by dark blue lines. Interactions not fully depending on the EH domain are shown with dashed lines. Additional interactions, derived from the BioGRID database (http://thebiogrid.org/) and from the literature, are shown by red lines. The picture was initially generated using the Osprey software [96] and then edited with Adobe Illustrator.

The physical and functional connections in the EH network of the nematode are reported in schematic form in Figure 5 and in an extended form in Figure S4; in addition, we report a number of characteristics of the identified EH interactors as obtained from literature searches and Wormbase (Table 2 and Table S1). We identified 26 interactors of EH domains by Y2H and validated a majority of them through *in vitro* binding assays and by genetic analysis (as shown synoptically in Figure S4).

**Table 2:** Characteristics of EH interactors.

EH interactor	Human Ortholog^1^	EH interaction previously identified^2^	Expression pattern^3^	Functional category^4^
EPN-1	EPN1	ITSN-1, EHS-1 [63]	Ubiquitous	END,TRA
LIN-10	APBA1/2	EHS-1, *itsn*-1 [98]	NS, INT,BWM	TRA
DAB-1	DAB1	*itsn*-1, *ehs*-1 [63]	VPC,VPC-de	END
F15C11.2	UBQLN1	ITSN-1-EH [63]	INT,PHA,HYP	END,UB/DEG
CAS-1	CAP1	None	N/A	ACT
PQN-32	-	None	N/A	UNKN
AIN-2	TNRC6A/	None	Ubiquitous	miRNA
K04H4.2	-	None	N/A	MET(?)
ALX-1	PDCD6IP	ITSN-1-EH [63], RME-1-EH [39]	Ubiquitous	END,TRA,AP
T05E7.5	-	None	N/A	UNKN.
TFG-1	TFG	None**	EMBR	AP,SIGN
F46H5.7	-	ITSN-1-EH [63]	N/A	UNKN
FLH-1	-	None	EMBR	TRAN,miRNA
Y37E3.11	PCYT2	None	N/A	MET
TAG-208	SORBS3	None	N/A	ACT
D1081.7	-	None	N/A	UNKN
BATH-42	SPOP*	None	NS, PHA, VM	UB/DEG/AP
BE0003N10.3	FBX11*	None	N/A	UB/DEG
T05F1.4	-	None	N/A	UNKN
SEL-5	AAK1	None	RS, VM	END
PCN-1	PCNA	None	N/A	REPL,REPA
VAB-19	KANK3*	None	EMBR, EPI	ACT
ALH-9	ALDH7A1	None	EMBR	MET
M03A8.3	-	None	N/A	UNKN
F23B12.5	DLAT	None	NS, INT ,PHA	MET
T23G11.7	VTA1	None	N/A	TRA

Some characteristics of EH-interactors. Additional information is in Table S1.

1 Human ortholog were identified through NCBI Homologene or by BLAST searches. (-) indicates that no human orthologue is immediately apparent; (*) indicates putative ortholog (best guess).

2 Previously known interactions between EH-containing proteins and EH-interactors were obtained from Wormbase or by literature search (indicated by the appropriate references). When the EH-containing protein is indicated (e.g. EHS-1), the physical interaction with the interactor has been described; when the gene is indicated (e.g. *ehs*-1), the genetic interaction between the genes has been described. (**); in the case of TFG-1, an interaction with the SH3 domains of ITSN-1 was described, by Y2H [63], and TFG-1 was identified by mass-spec in anti-ITSN-1 immunoprecipitates [63].

3 The expression patterns in *C. elegans* were derived from Wormbase. NS, nervous system; INT, intestine; PHA, pharynx; BWM, body wall muscles; VPC, vulval precursor cells; VPC-de, VPC descendants; HYP, hypodermis; EMBR, expressed during embryogenesis; VM, vulval muscle; RS, reproductive system; EPI, epidermis; N/A, not annotated.

4 Functional categories were derived from Wormbase, from the Gene Ontology database, from literature data or inferred from functions of the human homologues. END, endocytosis; TRA, membrane and vesicular traffic; UB/DE, ubiquitin system and protein degradation; ACT, actin dynamics; miRNA, miRNA function; MET, metabolism; AP, apoptosis; SIGN, signaling; TRAN, transcription; REPL, DNA replication; REPA, DNA repair; UNKN, unknown. (?) indicates hypothetical function.

We cannot be certain that we have identified all EH-interacting proteins. Few hypothetical interactors, as for example the synaptojanin homologue UNC-26, were unable to interact with the EH baits, even when directly tested. This might be due to “real” lack of interaction or to technical reasons. For instance, the absence – in the EH constructs used for the screening – of regions outside of the EH domain required to assist some EH-NPF interactions might have yielded a false negative result. It should also be mentioned that the nature of our screening does not allow for stringent conclusions in terms of affinity of the detected interactions. It is known that several variables affect the affinity and the selectivity of EH-NPF interactions, such as the amino acid composition of NPF surrounding regions [9], [10], [20], the presentation of the NPF tripeptide at the protein surface [66] or the presence of multiple NPF motifs ([23]; as a case in point, mammalian synaptojanin displays 3 NPF motifs, while UNC-26 has a unique NPF). Thus, low affinity interactions might have escaped our detection, but might still have relevance *in vivo*, if the local concentrations of the interactors are sufficiently high.

Notwithstanding the above considerations, a number of controls (described in the text above) support the notion that we should have obtained a near complete representation of the EH interactome for EHS-1, ITSN-1 and REPS-1. Conversely, we may have missed a number of interactions for the EH of RME-1, because of the nature of our screening. It has been shown that homo/hetero-oligomerization of EHD proteins is important for optimal binding to NPF-containing proteins [59], [67], [68], a condition that most likely was not achieved under the conditions of our initial Y2H screening, thus preventing the isolation of strong specific interactors. This is further supported by the fact that the EH domain of RME-1/EHD proteins, located in the carboxyl-terminal of the proteins, has a strong binding preference for NPF motifs followed by acidic residues [38], [69]. None of the proteins identified in our Y2H screens show an acidic consensus surrounding the NPF motif, suggesting that the RME-1 EH binding proteins we identified are probably promiscuous interactors. Indeed, the described interaction between AMPH-1 (amphiphysin) and RME-1, which was previously shown to be functionally relevant [40], was not identified in our screening. Regardless of the conditions of screening, it is of note that 14 of the 26 genes encoding for EH-interactors displayed genetic interactions with at least one gene encoding an EH-containing protein. This is remarkable, considering that only one phenotype (aldicarb sensitivity) was analyzed. While a number of these interactions (6 of 26) were already known, either in nematodes or in mammals (see Table 2 and Table S1), the others (20 of 26) are described here for the first time (Table 2): together, these interactions define the physical and functional landscape of the EH network at the organismal level in the nematode.

As shown in Figure 5, the most evident feature of the EH network is its involvement in endocytosis, traffic, and actin dynamics. These results confirm the role of the EH network in orchestrating processes in which coordination between the machineries of intracellular traffic and actin remodeling are required. This function is evolutionarily conserved: it has been confirmed in a number of high-resolution studies in mammals [69]–[75], and also by a virtual reconstruction of the EH network in yeast, which we performed by exploiting a number of publicly available interaction data and published high-throughput screens in *S. cerevisiae* (Figure S5).

At the biological level, the EH network seems to play a major role in neurotransmission in the nematode, as supported by the finding that RNAi of the majority of EH interactors (16 of 26) affected aldicarb sensitivity either in a WT background or in EH-containing proteins mutant strains (Figure 4A). While these results can probably be interpreted in the framework of the known participation of EH-containing proteins to the process of synaptic vesicle recycling [45], [63], [64], through the mentioned connections with endocytosis/traffic and actin dynamics, there is reason to postulate a wider involvement of the EH network in neurotransmission. In particular, the involvement of the EH network in the physiological regulation of the nervous system might also be mirrored by its putative subversion in pathological conditions. Indeed, some of the mammalian homologues of the EH-interacting proteins we identified in the nematode have been implicated in Alzheimer's disease (AD). DAB1 (*dab-1*), ubiquilin1 (F15C11.2) and Mint1 (*lin-10*) all bind the amyloid precursor protein (APP) and regulate β-amyloid (Aβ) production [76]-[81]. So far, EH-containing proteins have not been implicated in AD; however the recognized relevance of endocytosis and trafficking of APP in the etiology of AD [82]–[84] suggests the possibility that this family of proteins, and in particular eps15 and intersectin that are highly expressed in neurons, could participate, together with the identified partners, in AD pathology via altered APP endocytosis and trafficking.

A number of other “territories” of cellular activity are also intersected by the EH network (Figure 5 and Table 2). These include metabolism, signal transduction, apoptosis, and control of protein stability and/or activity through ubiquitination. While a detailed analysis of all EH interactors is impossible here, we would like to comment on two, partially overlapping, emerging features of the network: the potential involvement in i) nuclear functions, and ii) miRNA biogenesis and activity. The first case is suggested by the interaction of EH-containing proteins with the transcription factor FHL-1 (see also below), and with the PCN-1 protein (PCNA in mammals) that is involved in DNA replication and repair [85]. While these interactions need further validation and confirmation of their relevance, they are in line with the reported presence of EH-containing and EH-binding proteins, such as Eps15 or epsin [47], [49], in the nucleus of mammalian cells. The nuclear localization of these latter proteins is itself suggestive of a wider connection between endocytosis (or endocytic proteins) and nuclear functions, whose biological significance remains largely to be ascertained [50], [53], [86], [87].

The connection between the EH network and miRNA activity might impinge on at least two levels of regulation. On one level, miRNA transcription is regulated by FLH-1, which we have identified as an EH interactor. This protein belongs to the family of Zn-finger FLYWCH transcription factors that includes FLH-1, FLH-2, and FLH-3, and it has been shown to bind to the promoters of several nematode miRNA genes, and to repress their transcription [88]. FLH-1 is required for transcription of a set of miRNAs expressed specifically in the nervous system [88], further reinforcing the role of the EH network in neuronal functions. On another level, the EH-interactor AIN-2 (GW182 in mammals), together with Argonaute (Ago) proteins, constitutes the core of the so-called miRISC complex (miRNA-induced silencing complex), which associates with miRNAs for recognition of specific target mRNAs. miRISC controls the translational efficiency and/or the stability of mRNAs [89], [90]. In *C. elegans*, the repression of translation initiation also requires the GW182 proteins AIN-1 and AIN-2, and this mechanism operates on several mRNAs targeted by different miRNAs [91], [92].

The role of the EH network on miRNA activity also remains to be defined by future high-resolution studies. It should be viewed, however, in the context of the emerging liaison between the endocytic machinery and the control of miRNA function. Components of miRISC, including AGO proteins and GW182, are enriched in endosomes and MVBs [93]–[95]. The association of the EH network in this context has functional significance, since blocking the formation of MVBs from early endosomes decreases miRISC activity. Conversely, inhibiting the fusion of MVBs with the lysosome, and thereby reducing the clearance of miRISC through lysosomal degradation, increases miRISC activity. These results are compatible with a model in which the MVB membrane is a platform for the assembly of miRNA processing complexes [93]–[95] and provides a possible framework to interpret the involvement of components of the EH-network, whose participation in intracellular trafficking processes is well established.

## Supporting Information

Figure S1 **EH-containing proteins in various species.** At least four families of EH-containing proteins are present in *C. elegans*, *D. melanogaster* and *H. sapiens*: EPS15/EHS-1, ITSN/DAP160, EHD/PAST-1/RME-1, and REPS. A fifth family, represented by γ-synergin in *H. sapiens*, is not present in flies. In worms, the protein R10E11.6 might be a homologue of γ-synergin; however, the region of R10E11.6 displaying homology to the EH domain (indicated by a grey box) does not show binding properties, as shown in this study. In the yeast *S. cerevisiae*, the homology of EH-containing proteins to the families present in other species is much less clear. While Ede1p most likely constitutes the orthologue of the EPS15 family (harboring three EH domains, a coiled coil and a Ubiquitin binding domain), the other four yeast EH-containing proteins – Pan1p, Tax4p, Irs4p, and End3p – show less evident homology and conservation of functional domains with the nematode/fly/mammal families of EH-containing proteins. However, they can be assigned to one or another family on the basis of domain organization (EH domain at the C-terminus for the EHD/PAST/RME family and Tax4p and Irs4p) or as a function of their biological roles (as for Pan1 and Intersectin which are directly involved in the process of actin polymerization). The known functional domains of the various proteins are indicated.
(TIF)

Figure S2 **Alignment of EH domains of selected human (Hs) and nematode (Ce) proteins.** Secondary structure, as determined experimentally for the EH2 domain of human Eps15, is depicted above the alignment [1]. Position of residues in canonical EF-hands is indicated at the bottom of the alignment by pink boxes. The asterisk indicates the position of the proline residue in the EH domain of γ-synergin, where an aspartic acid is usually found. The red arrows point to the conserved Leucine and Tryptophan residues that were mutagenized to Alanine in the LWA mutants.
(TIF)

Figure S3 **Representative images of the** ***in vitro*** **binding assays shown in** **Figure 2** **of the main text.** EHS-1/ITSN-1/REPS-1/RME-1^LWA^ are mutant proteins containing point mutations that abolish the binding properties of the EH domains (see Figure S2 for the position of the mutations).
(TIF)

Figure S4 **A synopsis of all results obtained in the analysis of the EH-interactors is presented.** Data are extracted from the experiments shown in Figure 1, 2, and 4 of the main text. Note that bindings with efficiency <5% were considered as negative. The interaction between TAG-208 and REPS-1 is shown as “negative” in the IVB assay, since it did not depend directly on the EH domain of REPS-1, see Figure S3.
(TIF)

Figure S5 **The EH network in yeast.** An interaction diagram is shown representing *S. cerevisiae* EH proteins (red circles) together with their interactors (blue circles); the interactors are further grouped into functional categories. Interaction data were derived from the BioGRID database (http://thebiogrid.org/) and from literature. Not all interactions can be unequivocally attributed to EH-mediated contacts, since most of the data come from yeast two-hybrid screening experiments performed with full-length proteins. The picture was initially generated using the Osprey software [2], and then edited with Adobe Illustrator. Functional categories were derived as in Figure 5 of the main text.
(TIF)

Table S1 **Some characteristics of EH-interactors are reported. This Table represents an extended version of** **Table 2** **of the main text.** ^1^ Human orthologues were identified through NCBI Homologene or by BLAST searches. (-) indicates that no human orthologue is immediately apparent; (*) indicates putative orthologue (best guess). ^2^ Previously known interactions between EH-containing proteins and EH-interactors were obtained from Wormbase (WB) or through a literature search. When the EH-containing protein is indicated (e.g. EHS-1), the physical interaction with the interactor has been described; when the gene is indicated (e.g. *ehs*-1), the genetic interaction between the genes has been described. (**), in the case of TFG-1, an interaction with the SH3 domains of ITSN-1 was described, by Y2H [3], and TFG-1 was identified by mass-spec in anti-ITSN-1 immunoprecipitates [3]. ^3^ Descriptions were taken from Wormbase (biological processes) and manually edited. N/A, not annotated. ^4^ The expression patterns in *C. elegans* were derived from Wormbase. NS, nervous system; INT, intestine; PHA, pharynx; BWM, body wall muscles; VPC, vulval precursor cells; VPC-de, VPC descendants; HYP, hypodermis; EMBR, expressed during embryogenesis; VM, vulval muscle; RS, reproductive system; EPI, epidermis; N/A, not annotated. ^5^ Functions in mammals were derived from gene Ontology, NCBI (processes only). ^6^ Functional categories were derived from the Wormbase database, from the Gene Ontology database, from literature data or inferred from functions of the human homolog. END, endocytosis; TRA, membrane and vesicular traffic; UB/DEG, ubiquitin system and protein degradation; ACT, actin dynamics; miRNA, miRNA function; MET, metabolism; APO, apoptosis; SIG, signaling; TRAN, transcription; REPL, DNA replication; REPA, DNA repair; UNKN, unknown. (?) indicates hypothetical function.
(DOCX)
