# Supplementary figures and images for: A Snapshot of the Physical and Functional Wiring of the Eps15 Homology Domain Network in the Nematode
Source: PLoS One. 2013 Feb 12;8(2):e56383. doi: 10.1371/journal.pone.0056383 (PMC3570524; doi:10.1371/journal.pone.0056383)

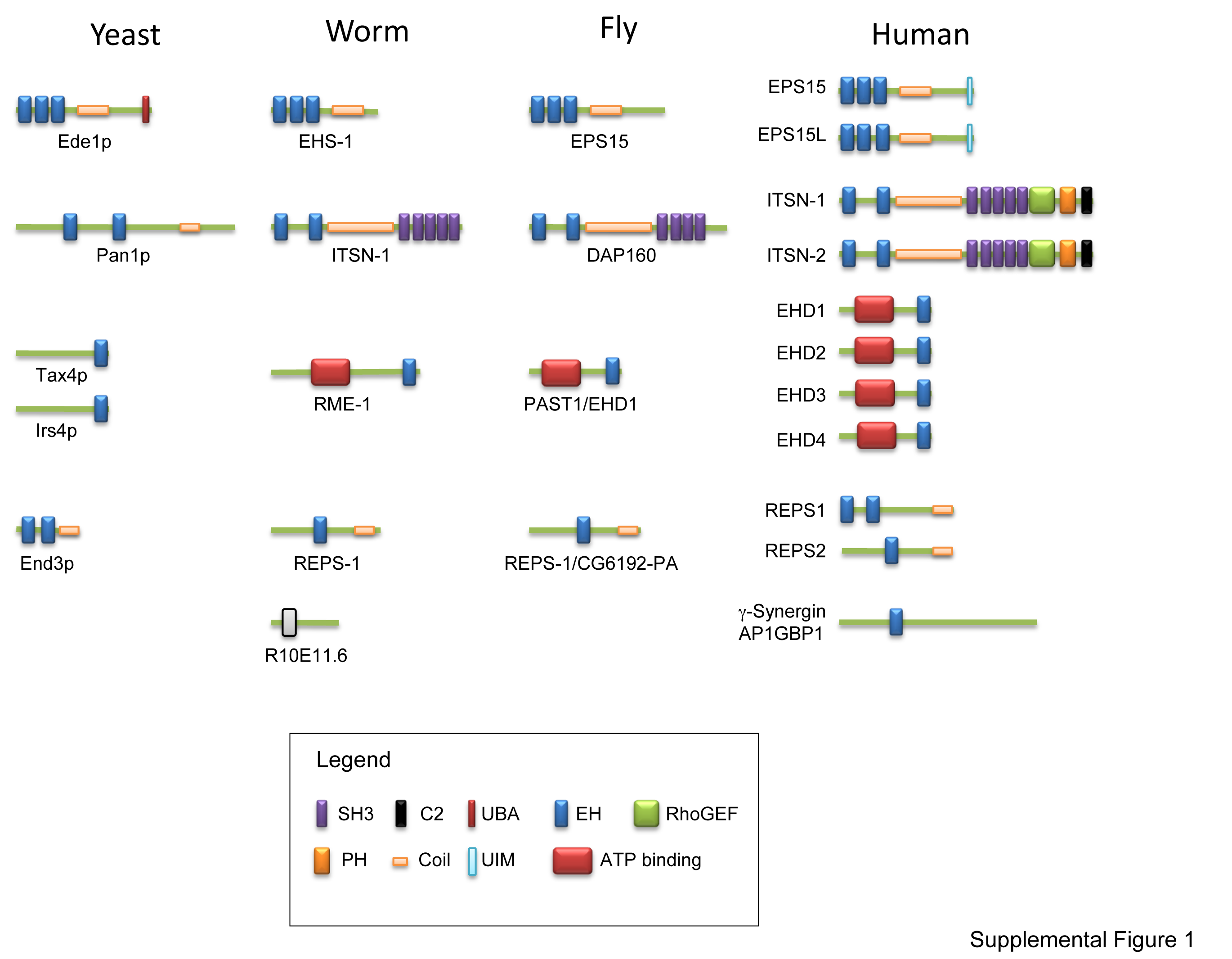

Supplement: Figure S1 — EH-containing proteins in various species. At least four families of EH-containing proteins are present in C. elegans, D. melanogaster and H. sapiens: EPS15/EHS-1, ITSN/DAP160, EHD/PAST-1/RME-1, and REPS. A fifth family, represented by γ-synergin in H. sapiens, is not present in flies. In worms, the protein R10E11.6 might be a homologue of γ-synergin; however, the region of R10E11.6 displaying homology to the EH domain (indicated by a grey box) does not show binding properties, as shown in this study. In the yeast S. cerevisiae, the homology of EH-containing proteins to the families present in other species is much less clear. While Ede1p most likely constitutes the orthologue of the EPS15 family (harboring three EH domains, a coiled coil and a Ubiquitin binding domain), the other four yeast EH-containing proteins – Pan1p, Tax4p, Irs4p, and End3p – show less evident homology and conservation of functional domains with the nematode/fly/mammal families of EH-containing proteins. However, they can be assigned to one or another family on the basis of domain organization (EH domain at the C-terminus for the EHD/PAST/RME family and Tax4p and Irs4p) or as a function of their biological roles (as for Pan1 and Intersectin which are directly involved in the process of actin polymerization). The known functional domains of the various proteins are indicated. (TIF) [file pone.0056383.s001.tif]

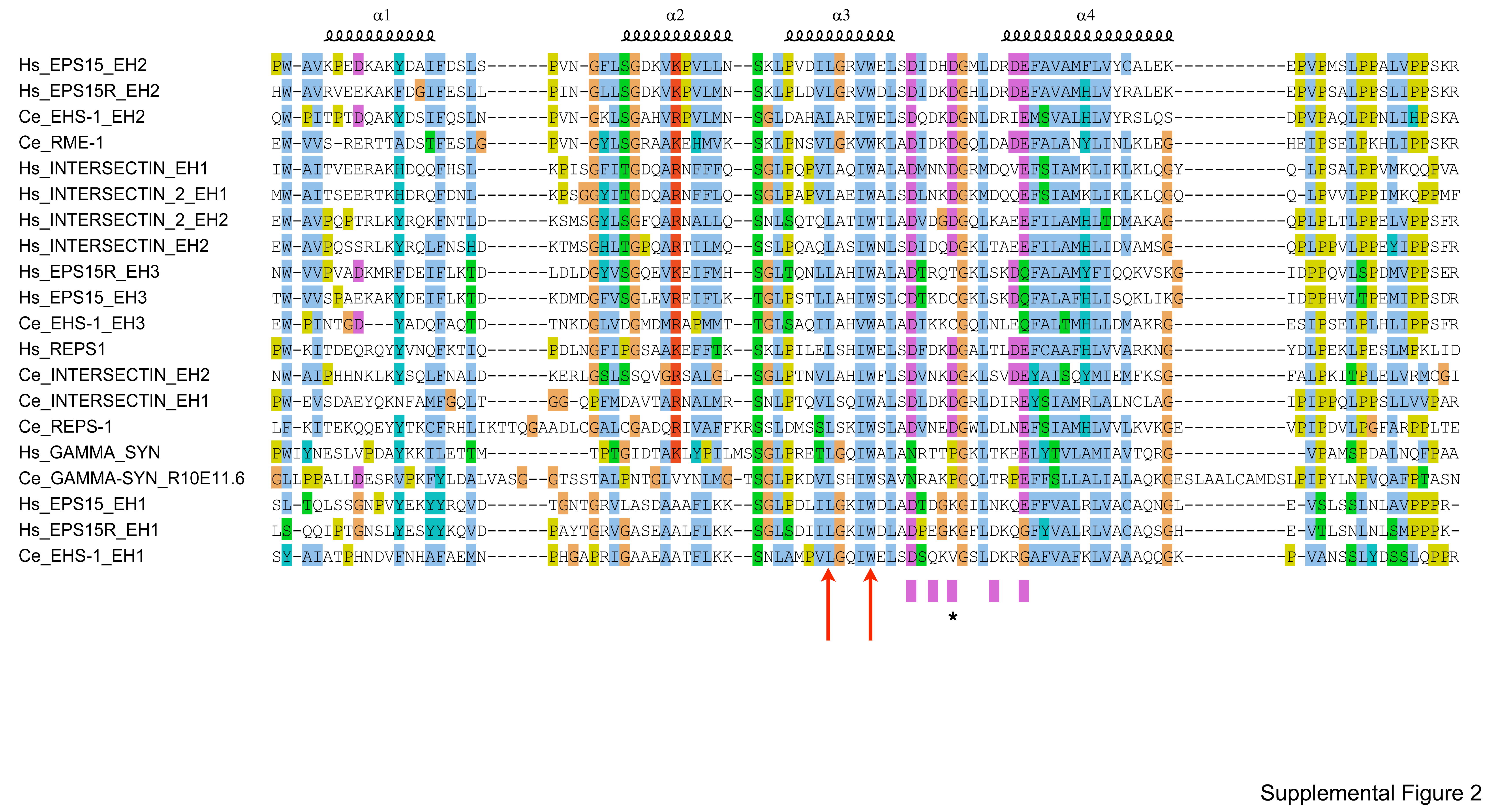

Supplement: Figure S2 — Alignment of EH domains of selected human (Hs) and nematode (Ce) proteins. Secondary structure, as determined experimentally for the EH2 domain of human Eps15, is depicted above the alignment [1]. Position of residues in canonical EF-hands is indicated at the bottom of the alignment by pink boxes. The asterisk indicates the position of the proline residue in the EH domain of γ-synergin, where an aspartic acid is usually found. The red arrows point to the conserved Leucine and Tryptophan residues that were mutagenized to Alanine in the LWA mutants. (TIF) [file pone.0056383.s002.tif]

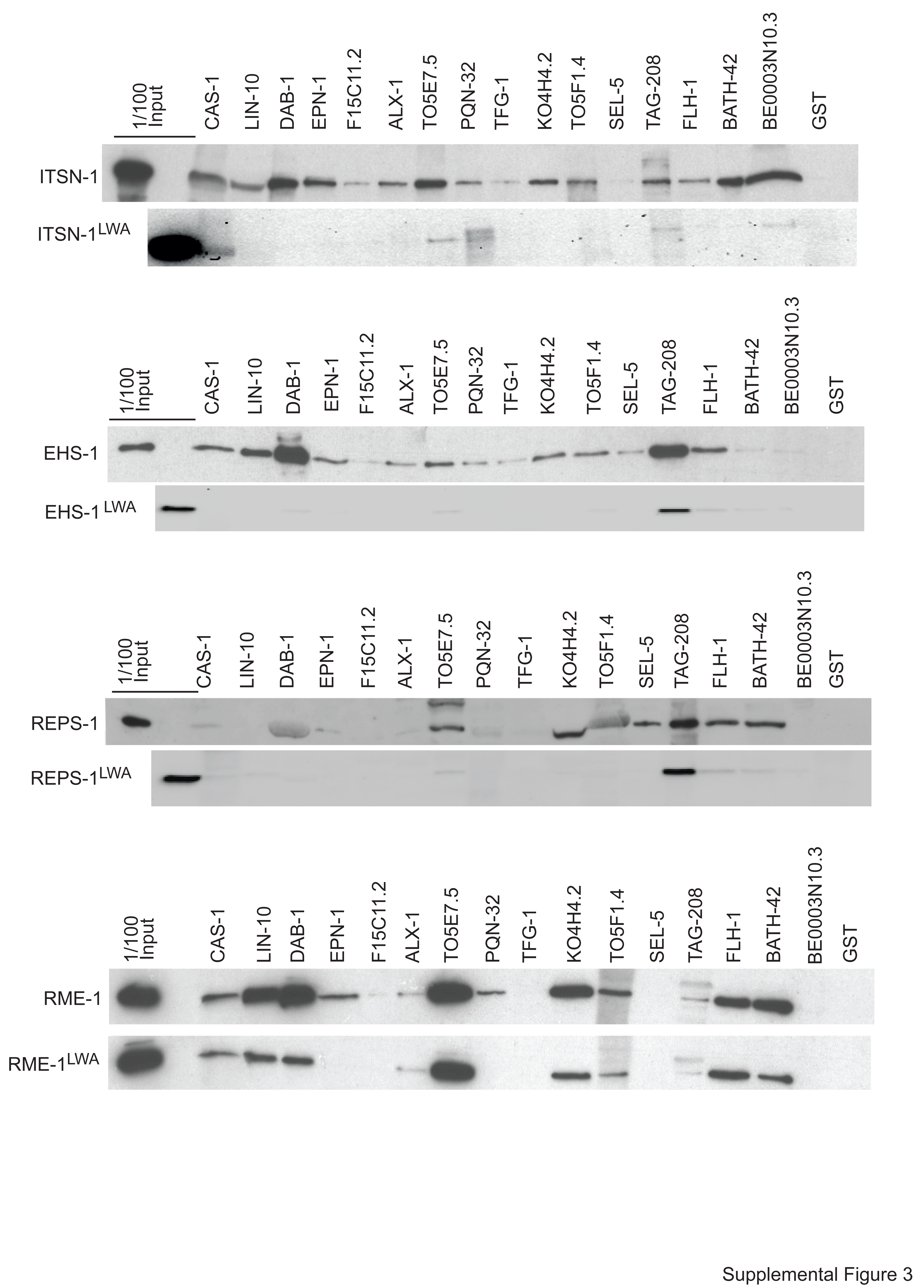

Supplement: Figure S3 — Representative images of the in vitro binding assays shown in Figure 2 of the main text. EHS-1/ITSN-1/REPS-1/RME-1LWA are mutant proteins containing point mutations that abolish the binding properties of the EH domains (see Figure S2 for the position of the mutations). (TIF) [file pone.0056383.s003.tif]

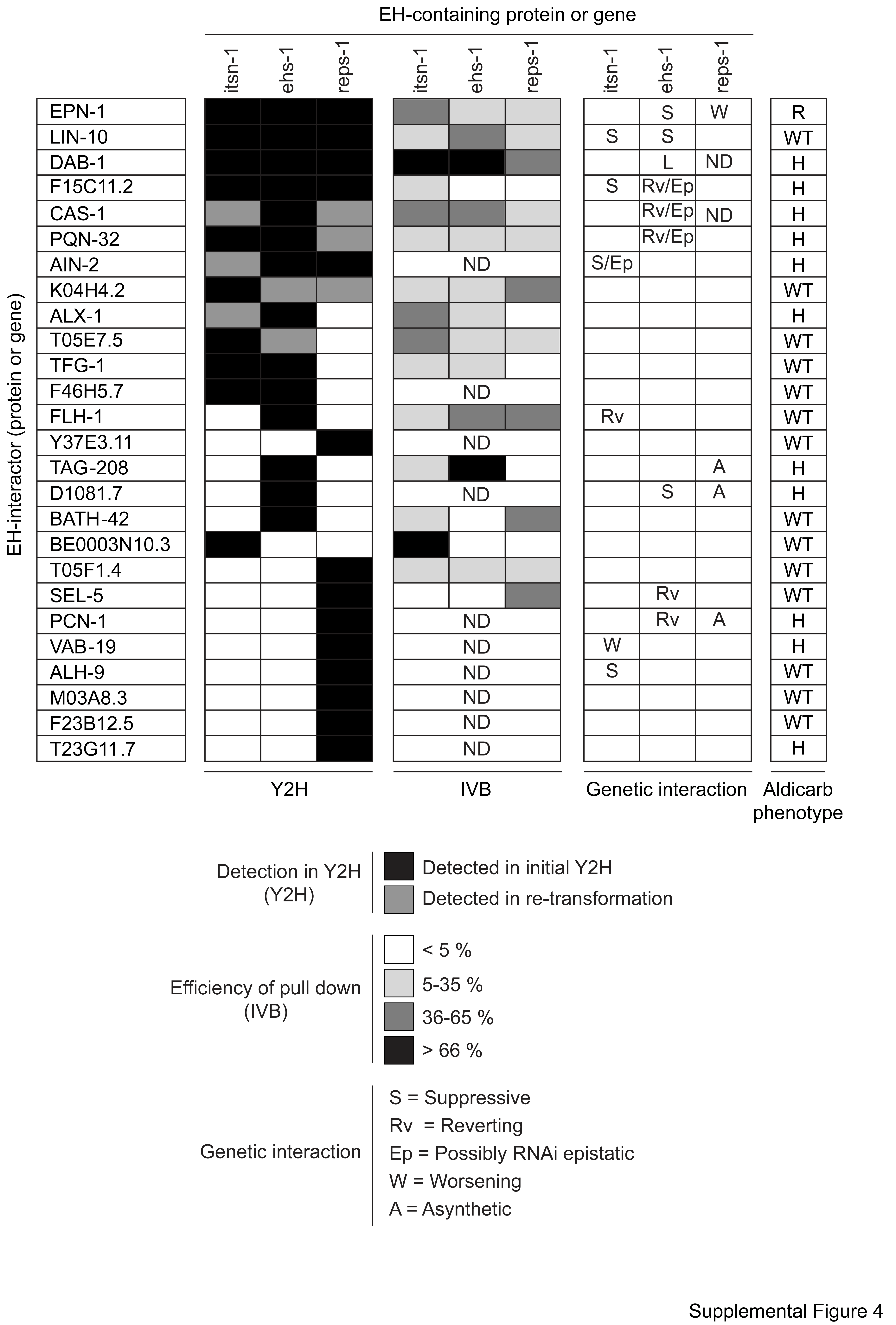

Supplement: Figure S4 — A synopsis of all results obtained in the analysis of the EH-interactors is presented. Data are extracted from the experiments shown in Figure 1, 2, and 4 of the main text. Note that bindings with efficiency <5% were considered as negative. The interaction between TAG-208 and REPS-1 is shown as “negative” in the IVB assay, since it did not depend directly on the EH domain of REPS-1, see Figure S3. (TIF) [file pone.0056383.s004.tif]

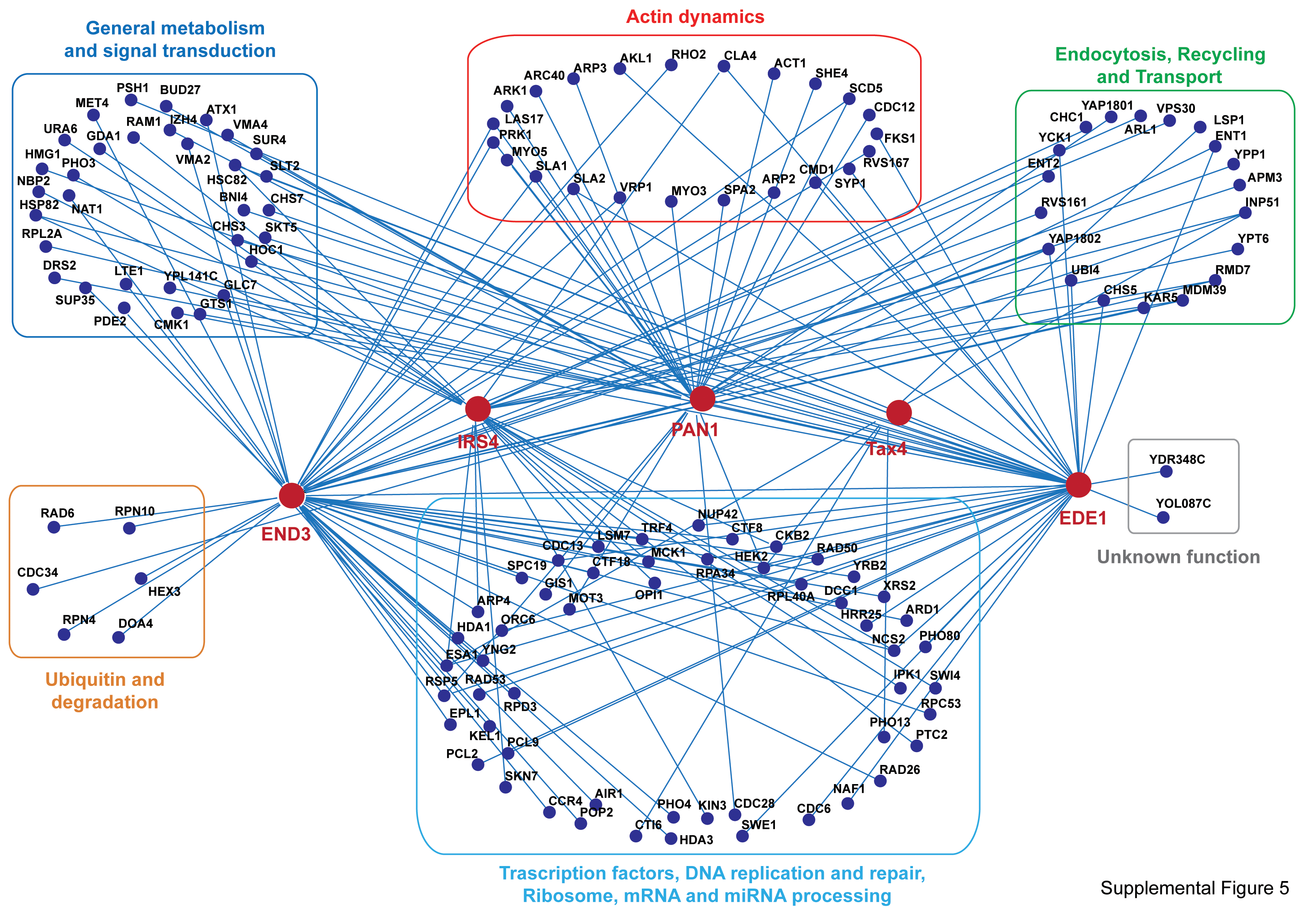

Supplement: Figure S5 — The EH network in yeast. An interaction diagram is shown representing S. cerevisiae EH proteins (red circles) together with their interactors (blue circles); the interactors are further grouped into functional categories. Interaction data were derived from the BioGRID database (http://thebiogrid.org/) and from literature. Not all interactions can be unequivocally attributed to EH-mediated contacts, since most of the data come from yeast two-hybrid screening experiments performed with full-length proteins. The picture was initially generated using the Osprey software [2], and then edited with Adobe Illustrator. Functional categories were derived as in Figure 5 of the main text. (TIF) [file pone.0056383.s005.tif]
