# Supplementary material for: A Snapshot of the Physical and Functional Wiring of the Eps15 Homology Domain Network in the Nematode
Source: PLoS One. 2013 Feb 12;8(2):e56383. doi: 10.1371/journal.pone.0056383 (PMC3570524; doi:10.1371/journal.pone.0056383)
Supplement: Table S1 — Some characteristics of EH-interactors are reported. This Table represents an extended version of Table 2 of the main text. 1 Human orthologues were identified through NCBI Homologene or by BLAST searches. (-) indicates that no human orthologue is immediately apparent; (*) indicates putative orthologue (best guess). 2 Previously known interactions between EH-containing proteins and EH-interactors were obtained from Wormbase (WB) or through a literature search. When the EH-containing protein is indicated (e.g. EHS-1), the physical interaction with the interactor has been described; when the gene is indicated (e.g. ehs-1), the genetic interaction between the genes has been described. (**), in the case of TFG-1, an interaction with the SH3 domains of ITSN-1 was described, by Y2H [3], and TFG-1 was identified by mass-spec in anti-ITSN-1 immunoprecipitates [3]. 3 Descriptions were taken from Wormbase (biological processes) and manually edited. N/A, not annotated. 4 The expression patterns in C. elegans were derived from Wormbase. NS, nervous system; INT, intestine; PHA, pharynx; BWM, body wall muscles; VPC, vulval precursor cells; VPC-de, VPC descendants; HYP, hypodermis; EMBR, expressed during embryogenesis; VM, vulval muscle; RS, reproductive system; EPI, epidermis; N/A, not annotated. 5 Functions in mammals were derived from gene Ontology, NCBI (processes only). 6 Functional categories were derived from the Wormbase database, from the Gene Ontology database, from literature data or inferred from functions of the human homolog. END, endocytosis; TRA, membrane and vesicular traffic; UB/DEG, ubiquitin system and protein degradation; ACT, actin dynamics; miRNA, miRNA function; MET, metabolism; APO, apoptosis; SIG, signaling; TRAN, transcription; REPL, DNA replication; REPA, DNA repair; UNKN, unknown. (?) indicates hypothetical function. (DOCX) [file pone.0056383.s006.docx]

**Table S1. Biological and functional characteristics of EH interactors**

| **EH**  **interactor** | **Human**  **Ortholog^1^** | **EH interaction previously identified^2^** | **Function in *C. elegans* from literature** | **Gene Ontology from WormBase^3^** | **Expression pattern in *C. elegans*^4^** | **Function in mammals**  **(Gene Ontology)^5^** | **Functional category^6^** |
| --- | --- | --- | --- | --- | --- | --- | --- |
| EPN-1 | EPN1 | ITSN-1, EHS-1 (WB).  ITSN-1-EH [3]. | - Potentiates Notch signaling during germline development [4]. | - Embryonic development ending in birth or egg hatching. - Lipid storage. - Receptor-mediated endocytosis. - Locomotion. - Positive regulation of growth rate. | Ubiquitous | - [Endocytosis](http://amigo.geneontology.org/cgi-bin/amigo/go.cgi?view=details&depth=1&query=6897). - Epidermal growth factor receptor signaling pathway. - [Negative regulation of epidermal growth factor receptor signaling pathway](http://amigo.geneontology.org/cgi-bin/amigo/go.cgi?view=details&depth=1&query=42059). | END, TRA |
| LIN-10 | APBA1/2 | EHS-1 (WB).  *itsn*-1 [5]. | - Controls proper GLR-1 receptor trafficking and localization in neurons [6]. - Controls basolateral localization of EGFR in the vulva precursor cells [7]. | - Asymmetric protein localization. - Nerve-nerve synaptic transmission. - Positive regulation of vulval development. - Sensory perception of touch. - Receptor clustering. | NS, INT, PHA, BWM | - Axon cargo transport. - Cell adhesion. - Intracellular protein transport. - Nervous system development. - Protein complex assembly. - Synaptic transmission. | TRA |
| DAB-1 | DAB1 | *itsn*-1, *ehs*-1 [3]. | - Controls VAB-1 receptor trafficking during oocyte meiotic maturation [8]. - Essential for yolk uptake by developing oocytes and required for endocytosis of molecules by coelomocytes [9]. - Mediates secretion of EGL-17, it functions as adaptors for vesicles containing LPRs and EGL-17 [10,11]. | - Oviposition. - Positive regulation of growth rate. - Protein secretion. - Vesicle-mediated transport. - Positive regulation of locomotion. - Receptor-mediated endocytosis. - Protein localization. - Embryonic development ending in birth or egg hatching. - Nematode larval development - Oocyte development. - Cell migration. - Molting cycle, collagen and cuticulin-based cuticle. | VPC, VPC-de. | - Cell differentiation. - [Multicellular organismal development](http://amigo.geneontology.org/cgi-bin/amigo/go.cgi?view=details&depth=1&query=7275). - [Nervous system development](http://amigo.geneontology.org/cgi-bin/amigo/go.cgi?view=details&depth=1&query=7399). | END |
| F15C11.2 | UBQLN1 | ITSN-1-EH [3]. | - Involved in ER stress and protein misfolding response [12]. - Suppresses polyglutamine-induced toxicity [13]. | N/A | INT, PHA, HYP | - [Apoptosis](http://amigo.geneontology.org/cgi-bin/amigo/go.cgi?view=details&depth=1&query=6915) regulation of protein ubiquitination. - [Response to hypoxia](http://amigo.geneontology.org/cgi-bin/amigo/go.cgi?view=details&depth=1&query=1666). | END, UB/DEG |
| CAS-1 | CAP1 | None |  | - Cytoskeleton organization. - Cell morphogenesis. | N/A | - [Actin cytoskeleton organization](http://amigo.geneontology.org/cgi-bin/amigo/go.cgi?view=details&depth=1&query=30036). - [Activation of adenylate cyclase activity](http://amigo.geneontology.org/cgi-bin/amigo/go.cgi?view=details&depth=1&query=7190). - [Ameboidal cell migration](http://amigo.geneontology.org/cgi-bin/amigo/go.cgi?view=details&depth=1&query=1667). - [Axon guidance](http://amigo.geneontology.org/cgi-bin/amigo/go.cgi?view=details&depth=1&query=7411). - [Blood coagulation](http://amigo.geneontology.org/cgi-bin/amigo/go.cgi?view=details&depth=1&query=7596). - [Cell morphogenesis](http://amigo.geneontology.org/cgi-bin/amigo/go.cgi?view=details&depth=1&query=902). - [Establishment or maintenance of cell polarity](http://amigo.geneontology.org/cgi-bin/amigo/go.cgi?view=details&depth=1&query=7163). - [Platelet activation](http://amigo.geneontology.org/cgi-bin/amigo/go.cgi?view=details&depth=1&query=30168). - [Platelet degranulation](http://amigo.geneontology.org/cgi-bin/amigo/go.cgi?view=details&depth=1&query=2576). - [Receptor-mediated endocytosis](http://amigo.geneontology.org/cgi-bin/amigo/go.cgi?view=details&depth=1&query=6898). - [Signal transduction](http://amigo.geneontology.org/cgi-bin/amigo/go.cgi?view=details&depth=1&query=7165). | ACT |
| PQN-32 | - | None |  | N/A | N/A |  | UNKN. |
| AIN-2 | TNRC6A/GW182 | None | - Controls translational repression and mRNA degradation [14,15]. | - Negative regulation of translational initiation. - Nuclear-transcribed mRNA catabolic process. | Ubiquitous | - RNA binding. - Nucleotide binding. - [Gene silencing by RNA](http://amigo.geneontology.org/cgi-bin/amigo/go.cgi?view=details&depth=1&query=2576). - Negative regulation of translation involved in gene silencing by miRNA. - Regulation of translation. | miRNA |
| K04H4.2 | - | None | - Involved in axon guidance [16]. | - Nematode larval development. - Growth. - Locomotion. - Positive regulation of growth rate. - Chitin metabolic process. | N/A |  | MET (?) |
| ALX-1 | PDCD6IP | ITSN-1-EH [3].  RME-1-EH [17]. | - Controls protein trafficking during vulva development [18]. - Controls sorting of endocytosed integral membrane proteins [17]. | - Regulation of protein catabolic process. - Endocytic recycling. - Endosome organization. - Protein localization in organelle. - Protein catabolic process. | Ubiquitous | - [Apoptosis](http://amigo.geneontology.org/cgi-bin/amigo/go.cgi?view=details&depth=1&query=6915). - [Cell cycle](http://amigo.geneontology.org/cgi-bin/amigo/go.cgi?view=details&depth=1&query=7049). - [Cell division](http://amigo.geneontology.org/cgi-bin/amigo/go.cgi?view=details&depth=1&query=51301). - [Interspecies interaction between organisms](http://amigo.geneontology.org/cgi-bin/amigo/go.cgi?view=details&depth=1&query=44419). - [Protein transport](http://amigo.geneontology.org/cgi-bin/amigo/go.cgi?view=details&depth=1&query=15031). | END, TRA, APO |
| T05E7.5 | - | None |  | N/A | N/A |  | UNKN. |
| TFG-1 | TFG | None** | - Inhibits developmentally programmed cell death [19]. - Required for protein secretion in the germline [20]. | - Negative regulation of apoptosis. - Regulation of cell size. - Embryonic development ending in birth or egg hatching. - Regulation of cell growth. - Regulation of multicellular organism growth. - Nematode larval development. - Growth. - Locomotion. - Reproduction. - Body morphogenesis. - Receptor-mediated endocytosis. - Oviposition. - Molting cycle, collagen and cuticulin-based cuticle. | EMBR | - [Positive regulation of I-kappaB kinase/NF-kappaB cascade](http://amigo.geneontology.org/cgi-bin/amigo/go.cgi?view=details&depth=1&query=43123). - [Signal transduction](http://amigo.geneontology.org/cgi-bin/amigo/go.cgi?view=details&depth=1&query=7165). | APO, SIGN. |
| F46H5.7 | - | ITSN-1-EH [3]. |  | N/A | N/A |  | UNKN. |
| FLH-1 | - | None | - Represses miRNA genes expression [21]. | - Negative regulation of gene-specific transcription. - Nematode larval development. - Embryonic development ending in birth or egg hatching. - Body morphogenesis | EMBR |  | TRAN, miRNA |
| Y37E3.11 | PCYT2 | None |  | N/A | N/A | - [Biosynthetic process](http://amigo.geneontology.org/cgi-bin/amigo/go.cgi?view=details&depth=1&query=9058). - [Phospholipid biosynthetic process](http://amigo.geneontology.org/cgi-bin/amigo/go.cgi?view=details&depth=1&query=8654). | MET |
| TAG-208 | SORBS3 | None |  | N/A | N/A | - [Cell adhesion](http://amigo.geneontology.org/cgi-bin/amigo/go.cgi?view=details&depth=1&query=7155). - [Muscle contraction](http://amigo.geneontology.org/cgi-bin/amigo/go.cgi?view=details&depth=1&query=6936). - [Positive regulation of cytoskeleton organization](http://amigo.geneontology.org/cgi-bin/amigo/go.cgi?view=details&depth=1&query=51495). - [Positive regulation of stress fiber assembly](http://amigo.geneontology.org/cgi-bin/amigo/go.cgi?view=details&depth=1&query=51496). | ACT |
| D1081.7 | - | None |  | N/A | N/A |  | UNKN. |
| BATH-42 | SPOP* | None | - Regulates maturation of nicotinic acetylcholine receptors [22]. | - protein binding | NS, PHA, VM | - [mRNA processing](http://amigo.geneontology.org/cgi-bin/amigo/go.cgi?view=details&depth=1&query=6397). | UB/DEG, APO |
| BE0003N10.3 | FBX11* | None |  | N/A | N/A | - [Peptidyl-arginine N-methylation](http://amigo.geneontology.org/cgi-bin/amigo/go.cgi?view=details&depth=1&query=35246). - [Protein modification process](http://amigo.geneontology.org/cgi-bin/amigo/go.cgi?view=details&depth=1&query=6464). - [Protein ubiquitination](http://amigo.geneontology.org/cgi-bin/amigo/go.cgi?view=details&depth=1&query=16567). - [Ubiquitin-dependent protein catabolic process](http://amigo.geneontology.org/cgi-bin/amigo/go.cgi?view=details&depth=1&query=6511). | UB/DEG |
| T05F1.4 | - | None |  | N/A | N/A |  | UNKN. |
| SEL-5 | AAK1 | None | - Participates in signaling events in vulva development [23]. - Involved in controlling longevity [24]. | - Determination of adult life span. - Embryonic development ending in birth or egg hatching. - Protein amino acid phosphorylation. | RS, VM | - [Protein phosphorylation](http://amigo.geneontology.org/cgi-bin/amigo/go.cgi?view=details&depth=1&query=6468). | END |
| PCN-1 | PCNA | None | - Required for embryogenesis [25]. | - Embryonic development ending in birth or egg hatching. - Cytokinesis. - Cell division. - Reproduction. - Regulation of DNA replication. | N/A | - [DNA repair](http://amigo.geneontology.org/cgi-bin/amigo/go.cgi?view=details&depth=1&query=6281). - [DNA replication](http://amigo.geneontology.org/cgi-bin/amigo/go.cgi?view=details&depth=1&query=6260). - [DNA strand elongation.](http://amigo.geneontology.org/cgi-bin/amigo/go.cgi?view=details&depth=1&query=6271) - [Involved in DNA replication](http://amigo.geneontology.org/cgi-bin/amigo/go.cgi?view=details&depth=1&query=6271) [G1/S transition of mitotic cell cycle](http://amigo.geneontology.org/cgi-bin/amigo/go.cgi?view=details&depth=1&query=82). - [Base-excision repair](http://amigo.geneontology.org/cgi-bin/amigo/go.cgi?view=details&depth=1&query=6284). - [Cell proliferation](http://amigo.geneontology.org/cgi-bin/amigo/go.cgi?view=details&depth=1&query=8283). - [Intracellular protein transport](http://amigo.geneontology.org/cgi-bin/amigo/go.cgi?view=details&depth=1&query=6886). - [Phosphatidylinositol-mediated signaling](http://amigo.geneontology.org/cgi-bin/amigo/go.cgi?view=details&depth=1&query=48015). - [Positive regulation of deoxyribonuclease activity](http://amigo.geneontology.org/cgi-bin/amigo/go.cgi?view=details&depth=1&query=32077). - [Regulation of DNA replication](http://amigo.geneontology.org/cgi-bin/amigo/go.cgi?view=details&depth=1&query=6275). - [Response to cadmium ion](http://amigo.geneontology.org/cgi-bin/amigo/go.cgi?view=details&depth=1&query=46686). - [Response to lipid](http://amigo.geneontology.org/cgi-bin/amigo/go.cgi?view=details&depth=1&query=33993) [telomere maintenance](http://amigo.geneontology.org/cgi-bin/amigo/go.cgi?view=details&depth=1&query=723). - [Transcription-coupled nucleotide-excision repair](http://amigo.geneontology.org/cgi-bin/amigo/go.cgi?view=details&depth=1&query=6283). | REPL, REPA |
| VAB-19 | KANK3* | None | - Required for epidermal embryogenesis [26]. | - Embryonic morphogenesis. - Actin cytoskeleton organization. - Embryonic development. - Cell-cell junction maintenance. - Protein localization. - Embryonic development ending in birth or egg hatching. - Body morphogenesis. - Molting cycle, collagen and cuticulin-based cuticle. - Positive regulation of growth rate. - Hermaphrodite genitalia development. | EMBR, EPI | - [Negative regulation of stress fiber assembly](http://amigo.geneontology.org/cgi-bin/amigo/go.cgi?view=details&depth=1&query=51497). | ACT |
| ALH-9 | ALDH7A1 | None |  | - Metabolic process. - Oxidation reduction. | EMBR | - [Cellular aldehyde metabolic process](http://amigo.geneontology.org/cgi-bin/amigo/go.cgi?view=details&depth=1&query=6081). - [Cellular nitrogen compound metabolic process](http://amigo.geneontology.org/cgi-bin/amigo/go.cgi?view=details&depth=1&query=34641). - [Lysine catabolic process](http://amigo.geneontology.org/cgi-bin/amigo/go.cgi?view=details&depth=1&query=6554)[.](http://amigo.geneontology.org/cgi-bin/amigo/go.cgi?view=details&depth=1&query=55114) - [Sensory perception of sound](http://amigo.geneontology.org/cgi-bin/amigo/go.cgi?view=details&depth=1&query=7605). | MET |
| M03A8.3 | - | None |  | N/A | N/A |  | UNKN. |
| F23B12.5 | DLAT | None |  | N/A | NS, INT, PHA, BWM | - [Acetyl-CoA biosynthetic process](http://amigo.geneontology.org/cgi-bin/amigo/go.cgi?view=details&depth=1&query=6085) - [Glycolysis](http://amigo.geneontology.org/cgi-bin/amigo/go.cgi?view=details&depth=1&query=6096). - [Metabolic process](http://amigo.geneontology.org/cgi-bin/amigo/go.cgi?view=details&depth=1&query=8152): [pyruvate.](http://amigo.geneontology.org/cgi-bin/amigo/go.cgi?view=details&depth=1&query=6090) - [Metabolic process](http://amigo.geneontology.org/cgi-bin/amigo/go.cgi?view=details&depth=1&query=6090): [regulation of acetyl-CoA.](http://amigo.geneontology.org/cgi-bin/amigo/go.cgi?view=details&depth=1&query=10510) - [Biosynthetic process from pyruvate](http://amigo.geneontology.org/cgi-bin/amigo/go.cgi?view=details&depth=1&query=10510). | MET |
| T23G11.7 | VTA1 | None |  | N/A | N/A | - [Cellular membrane organization](http://amigo.geneontology.org/cgi-bin/amigo/go.cgi?view=details&depth=1&query=16044). - [Endosome transport](http://amigo.geneontology.org/cgi-bin/amigo/go.cgi?view=details&depth=1&query=16197). - [Protein transport](http://amigo.geneontology.org/cgi-bin/amigo/go.cgi?view=details&depth=1&query=15031). | TRA |
